# Female *Drosophila melanogaster* respond to song-amplitude modulations

**DOI:** 10.1242/bio.032003

**Published:** 2018-04-17

**Authors:** Birgit Brüggemeier, Mason A. Porter, Jim O. Vigoreaux, Stephen F. Goodwin

**Affiliations:** 1 Centre for Neural Circuits and Behaviour, University of Oxford, Oxford OX1 3SR, UK; 2 AudioLabs, Fraunhofer-Institut für Integrierte Schaltungen, 91058 Erlangen, Germany; 3 Department of Mathematics, University of California, Los Angeles, Los Angeles, CA 90095, USA; 4 Mathematical Institute, University of Oxford, Oxford OX2 6GG, UK; 5 CABDyN Complexity Centre, University of Oxford, Oxford OX1 1HP, UK; 6 Department of Biology, University of Vermont, Burlington, VT 05405, USA

**Keywords:** *Drosophila*, Fruit flies, Courtship, Song amplitude structure, Communication signals

## Abstract

Males in numerous animal species use mating songs to attract females and intimidate competitors. We demonstrate that modulations in song amplitude are behaviourally relevant in the fruit fly *Drosophila*. We show that *Drosophila* *melanogaster* females prefer amplitude modulations that are typical of *melanogaster* song over other modulations, which suggests that amplitude modulations are processed auditorily by *D. melanogaster*. Our work demonstrates that receivers can decode messages in amplitude modulations, complementing the recent finding that male flies actively control song amplitude. To describe amplitude modulations, we propose the concept of song amplitude structure (SAS) and discuss similarities and differences to amplitude modulation with distance (AMD).

This article has an associated First Person interview with the first author of the paper.

## Abstract (precis)

**Summary:** We show that flies respond behaviourally to song amplitude structure; this observation suggests it may be a communication signal in *Drosophila*. We discuss our work in the broader context of other animals using amplitude modulations to communicate.

## INTRODUCTION

While courting, males can signal advantageous characteristics – such as fitness, agility, and strength – which may help females make mating decisions (Clutton-Brock and Albon, 1979; Elemans et al., 2008; Velez, 2013; Roemer, 2013). For example, female deer (Clutton-Brock and Albon, 1979), frogs (Velez, 2013), and crickets (Roemer, 2013) prefer males with large-amplitude calls. This suggests that the amplitude of courtship calls affects female receptivity. Modulation of amplitude can also convey relevant information. For example, vocal muscles control song production in starlings, and muscle activity modulates their song amplitude (Elemans et al., 2008); and these birds may be able to assess the muscle characteristics of males from their songs (Ritschard et al., 2010). Moreover, several animals modulate their courtship song amplitude with distance (‘amplitude modulation with distance’; AMD), and females may assess the distance to potential partners from these modulations (Velez, 2013; Brumm and Slater, 2006; Coen et al., 2016).

Male fruit flies court females by extending and vibrating one wing to produce a courtship song with species-specific characteristics (Bennet-Clark and Ewing, 1969). Song characteristics influence behavioural responses, including female receptivity (Bennet-Clark and Ewing, 1969; Eberl et al., 1997). Recently, Coen et al. (2016) showed that male flies actively modulate their song amplitude based on their distance from females. Specifically, males sing louder when a female is farther away. AMD is under motor-sensory control (Coen et al., 2016), and flies with low muscle power modulate their songs less than flies with normal muscle power. This suggests that amplitude modulations may convey relevant information – e.g. about the robustness of potential partners and the distance to them – to female flies. If true, females may respond to differences in amplitude modulations.

Coen et al. noted that AMD does not explain all amplitude modulations in songs. Physical limitations (e.g. muscle power output, wing hinge compliance, and thoracic tensions) and wing choice (Coen et al., 2016) may also contribute to amplitude modulations, but they are not taken into account by AMD. We introduce the novel concept of ‘song amplitude structure’ (SAS), which describes amplitude variations in songs (B. Brüggemeier, PhD thesis, University of Oxford, 2017). Song amplitude structure refers to an amplitude increase across a local amplitude peak followed by a decrease to a local minimum, inclusive of all fluctuations in song amplitude. Our work demonstrates that *Drosophila* *melanogaster* mate preferentially in response to song amplitude structure that is typical for their song over those with other amplitude modulations, suggesting that song amplitude structure is behaviourally relevant in *D. melanogaster*.

## RESULTS

### *D. melanogaster* females differentiate amplitude modulations

**Fig. 1.**
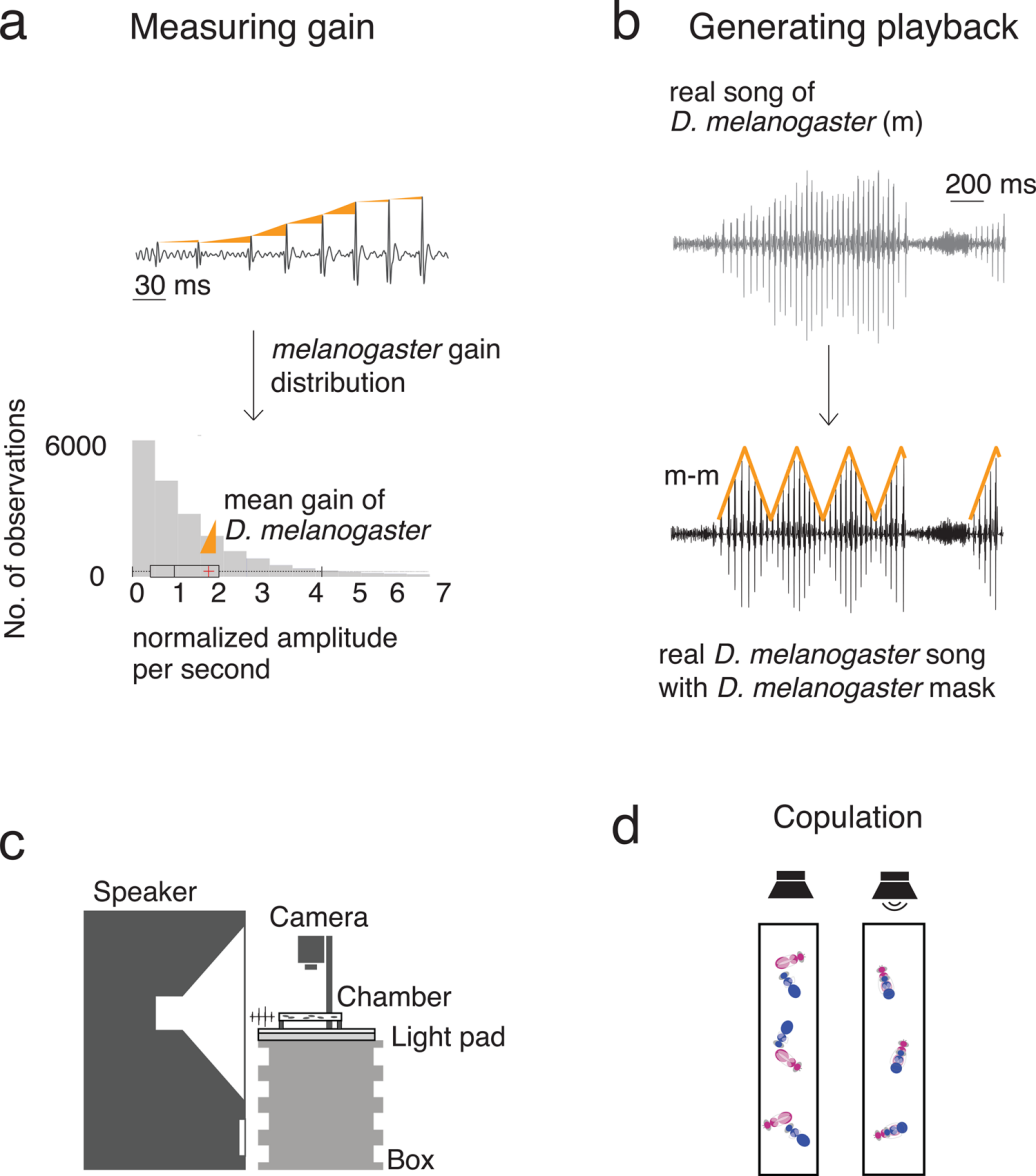
**Measuring and manipulating gain in fly songs and experimental setup for testing auditory responses to song amplitude structure in *Drosophila*.** (A) We measured the amplitude gain of pulses as the relative increase in amplitude of successive pulses. For example, suppose (using arbitrary units) that a pulse has an amplitude of 1 and is followed by a pulse with an amplitude of 2. The relative increase between those pulses is 2. (B) We created playback stimuli by masking 5 min of species-specific real song with a strain's mean gain envelopes. Songs of *D. melanogaster* exhibit species-specific characteristics (see Table 1), and our amplitude modulation does not alter the song parameters (see Fig. 3). The notation m-m refers to *D. melanogaster* song with *D. melanogaster* mean gain. (C) Schematic of our playback setup. (D) Schematic of our copulation assay. We muted males (shown in blue; females are in pink) by removing their wings and deafened them by removing their arista. Fly mating occurs more often during song playback (right) than during silence (left).

In Fig. 1A, we show amplitude gain in *D. melanogaster* song. We measured gain, and we examined whether female flies make mating decisions based on gain differences, analogous to females making mating decisions based on inter-pulse interval (IPI) differences (Bennet-Clark and Ewing, 1969; Ritchie et al., 1999; Vaughan et al., 2014). In Fig. 1B, we illustrate our playback stimuli design. See the section ‘Stimulus design’ in the Materials and Methods for a description of how we modified gain in our playback stimuli. To test whether female flies make mating decisions based on amplitude gain differences, we used copulation frequency in response to playback as a behavioural assay (Bennet-Clark and Ewing, 1969; Ritchie et al., 1999; Vaughan et al., 2014). For a schematic of our playback setup, see Fig. 1C; for an illustration of the copulation assay, see Fig. 1D. For our copulation assays, we deafened males by removing their arista and silenced them by removing their wings. Because deafened males do not respond to song playback (Vaughan et al., 2014; Kyriacou and Hall, 1982; Yoon et al., 2013), we interpret our results for copulation assays in terms of female auditory responses and mating decisions.

**Table 1.**
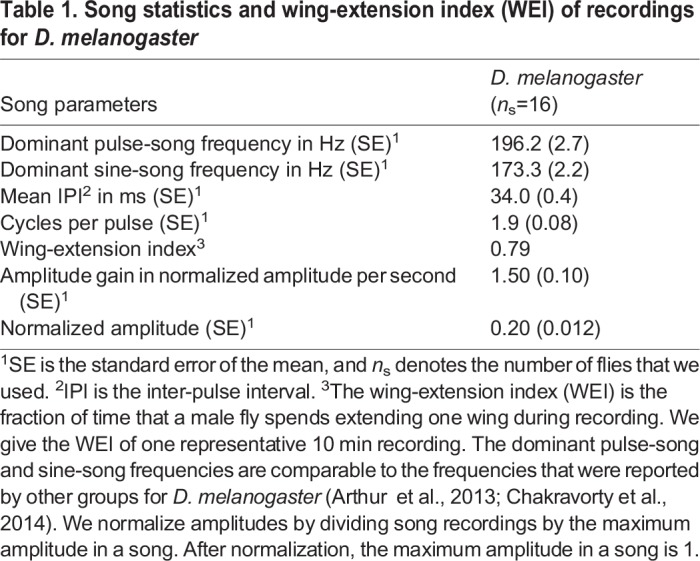
**Song statistics and wing-extension index (WEI) of recordings for *D. melanogaster***

**Fig. 2.**
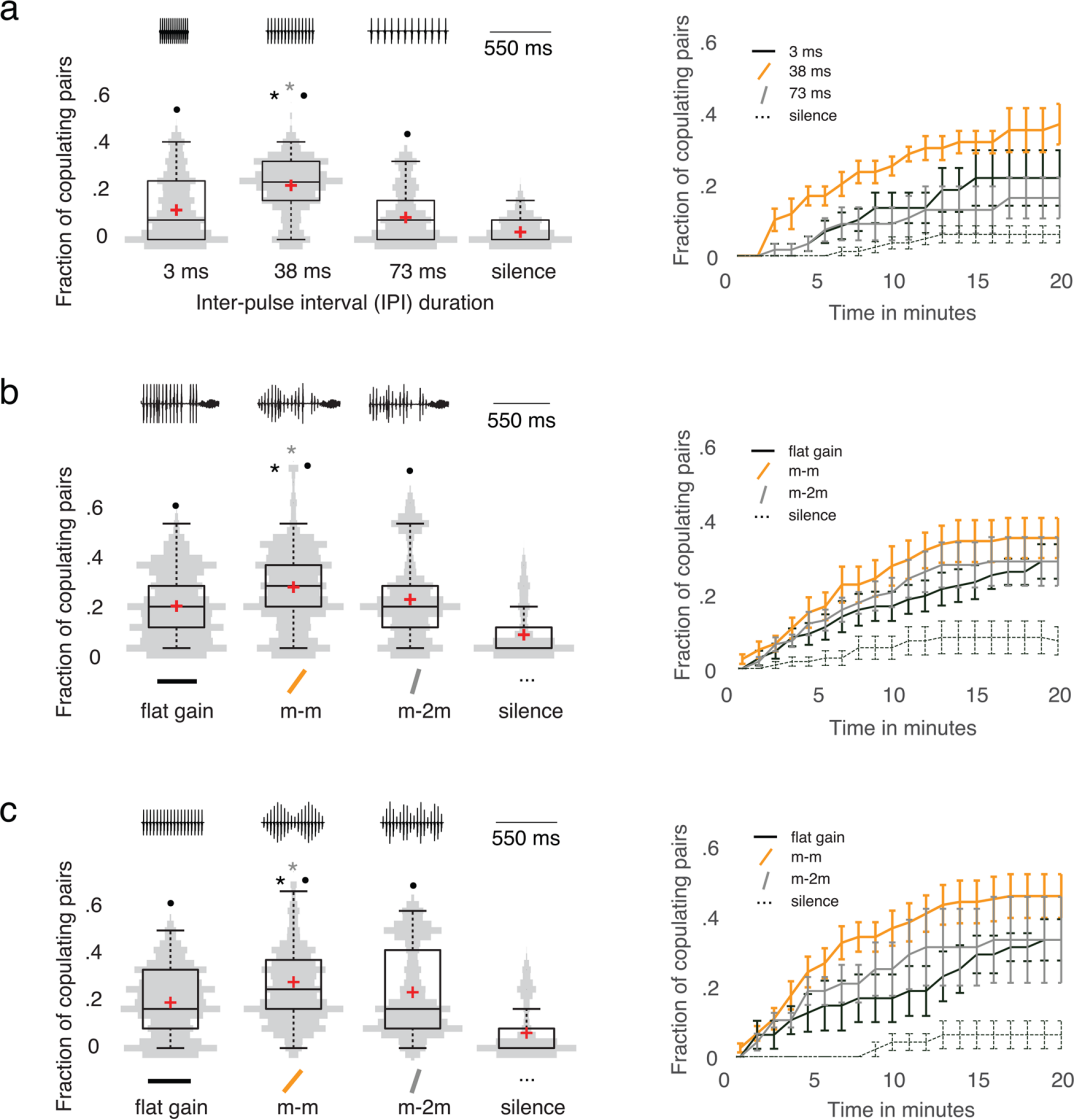
**Amplitude envelope preference of female *D. melanogaster*.** We present our data in two different forms: (left) violin distribution plots and (right) survivorship curves. (A) *D. melanogaster* CS females prefer their own strain's IPI duration (IPI = 38 ms) over shorter IPIs (3 ms), longer IPIs (73 ms), and the silence control condition. We calculated a *P*-value of *P*<0.0001 and an *F* statistic of *F* ≈ 34.51 in a one-way ANOVA test. There are *n*_c_=264 mixed-sex couples. To measure IPIs, we detected pulses in modulated playbacks automatically with FlySongSegmenter (Arthur et al., 2013) and then computed the distance between detected pulses. (B) *D. melanogaster* CS females preferred their own strain's song-amplitude-structure envelope (m-m) over flat gain, steeper gain (twice the *D. melanogaster* mean gain, which we denote by m-2m), and the silence control condition when we modulated song amplitude structure in a courtship song recording of a *D. melanogaster* male. We calculated *P*<0.0001 and *F*≈10.99 in a one-way ANOVA test, where *n*_c_=444 is the number of mixed-sex couples. (See Fig. 1B for an illustration of amplitude modulation in courtship-song recordings.) (C) *D. melanogaster* CS females preferred their own strain's amplitude envelope (m-m) over flat gain, steeper gain (m-2m), and the silence control condition when we modulated amplitude in an artificial *D. melanogaster* song. We calculated *P*<0.0001 and *F*≈18.33 in a one-way ANOVA with *n*_c_=228 mixed-sex couples. We show the distribution of the fraction of copulating pairs as grey kernel-density plots, which we mirror across the vertical axis and also show as box plots. A red cross indicates the mean, a horizontal line indicates the median, a box indicates the inter-quartile-range (IQR), and the whiskers indicate 1.5 × IQR. We calculated *P*<0.01 using two-sided Wilcoxon rank-sum (WR) tests, with Bonferroni correction for multiple testing; and we obtained *P*<0.025 using log-rank tests for comparing survival curves. The asterisks indicate a significant difference in the fraction of copulating pairs. The colour of the asterisks indicates which gain condition differs significantly from the others. The grey asterisks signifies that the grey condition (i.e. the m-2m envelope) differs significantly from the labelled condition (i.e. the *D. melanogaster* mean-gain envelope). The black asterisks indicate that the black condition (i.e. the flat gain) differs significantly from the labelled condition (i.e. the *D. melanogaster* mean-gain envelope). The black dots indicate that there is a significant difference in the fraction of copulating pairs between the labelled condition and the silence control; the *P*-value is *P*<0.01 (WR).

**Fig. 3.**
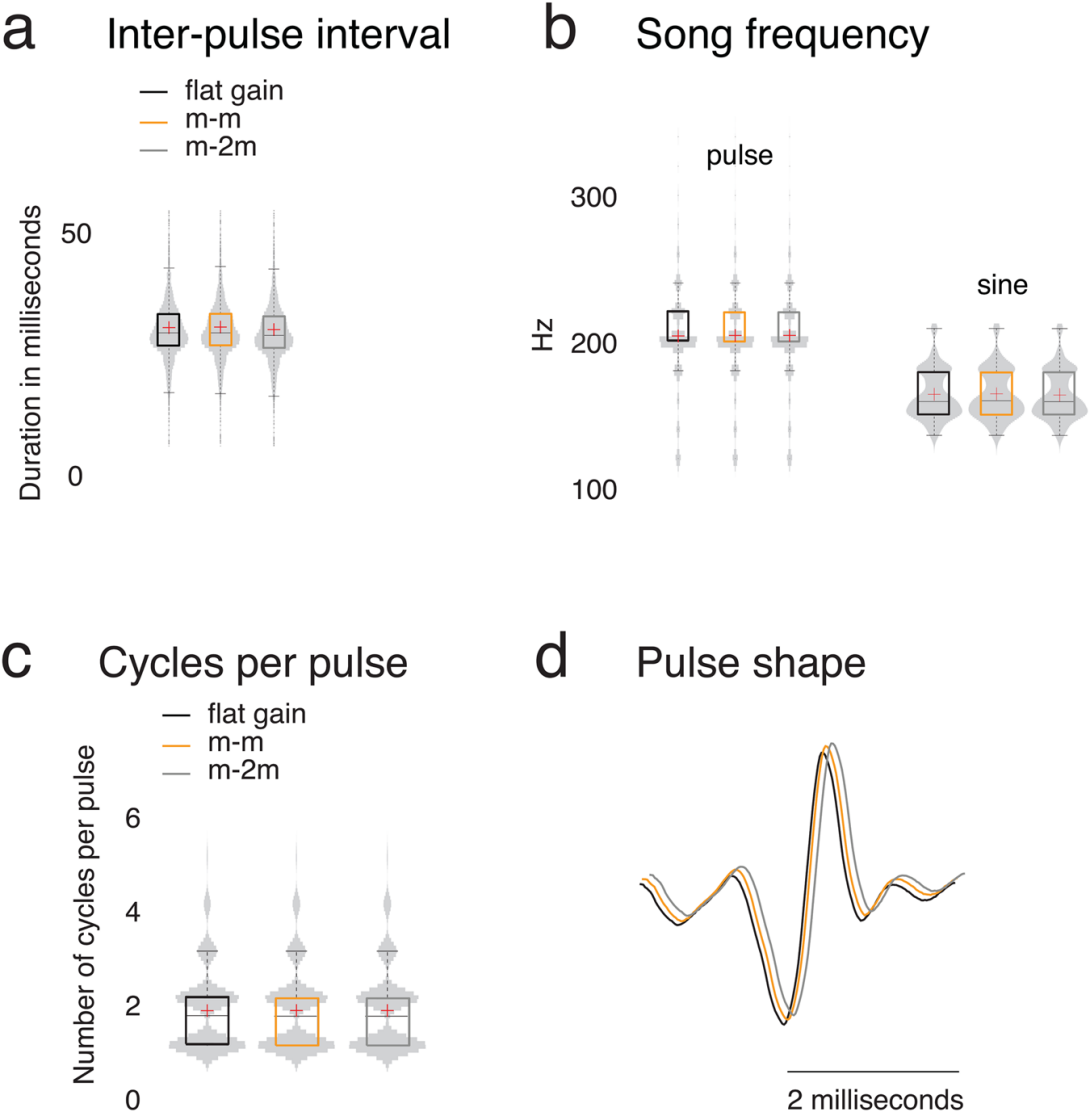
**Song parameters do not differ significantly across playback conditions.** (A) Inter-pulse interval (IPI) does not differ significantly across different playback conditions. The number *n*_p_ of detected pulses also does not differ significantly across different playback conditions for *D. melanogaster*. *D. melanogaster* song has *n*_p_=1870 for flat gain, *n*_p_=1873 for m-m, and *n*_p_=1869 for m-2m, where we recall that m-m refers to *D. melanogaster* song with *D. melanogaster* mean gain and m-2m refers to *D. melanogaster* song with twice the *D. melanogaster* mean gain. We show song statistics for each envelope condition (black for flat gain, orange for m-m, and grey for m-2 m) as light-grey kernel-density plots, which we mirror across the vertical axis and also show as box plots. A red cross indicates the mean, a horizontal line indicates the median, a box indicates the IQR, and the whiskers indicate 1.5 × IQR. (B) Neither pulse-song frequency nor sine-song frequency differ significantly across different playback conditions. We modulated pulse amplitude only, but we also present sine-song statistics, as sine song can influence fly behaviour (von Schilcher, 1976a). Our data suggest that sine song is similar across playback conditions, so differences in sine song are not sufficient to explain the behavioural differences that we observed. (C) Cycles per pulse (CPP) do not differ significantly across different playback conditions. (D) The mean pulse shape (computed as a pointwise mean) does not differ significantly across different playback conditions.

**Fig. 4.**
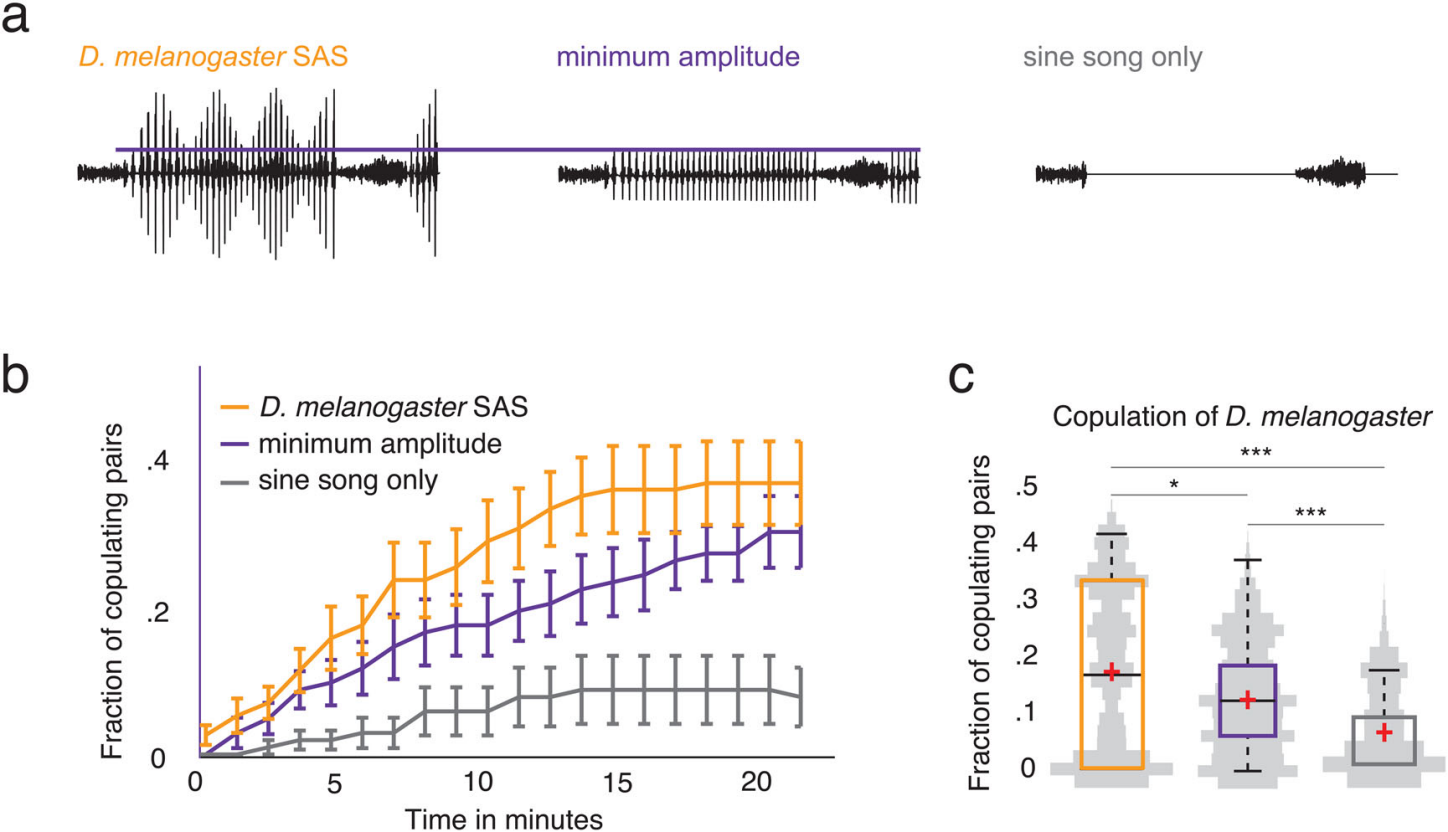
**Small-amplitude pulses in modulated songs arouse flies.** (A) We show m-m playback as a control to illustrate the amplitude level of pulses with minimum amplitude in the playbacks. To study whether flies hear pulses with small amplitudes in modulated songs, we generated playback with pulses set to the minimum amplitude that occurred in the m-m playback. If pulses at the minimum amplitude are not heard by flies, they should behave similarly in their responses to songs with pulses at minimum amplitude as in their responses to songs with no pulses (i.e. a sine-song-only condition). (B) Flies mate significantly more in response to songs with pulses at minimum amplitude (purple curve) than to songs with no pulses (grey curve). (C) The asterisks indicate significance levels: * signifies *P*<0.005 and *** signifies *P*<0.0005. In each case, we apply Bonferroni correction for multiple testing using a two-sided Wilcoxon signed-rank test. We show song statistics for each playback condition (orange for m-m, purple for minimum-amplitude pulses, and grey for sine-song only) as light-grey kernel-density plots, which we mirror across the vertical axis and also show as box plots.

In accordance with previous work (Bennet-Clark and Ewing, 1969; Vaughan et al., 2014), we find that *D. melanogaster* females prefer their species IPI over both longer and shorter IPI durations (see Fig. 2A). We examined whether *D. melanogaster* Canton-S (CS) females can differentiate their strain's amplitude gain versus ones with either flatter or steeper gains. To test female preferences for amplitude modulations, we conducted an experiment with four playback conditions: (1) a song with constant amplitude (flat gain; see Fig. 2B), (2) a song with the *D. melanogaster* mean-gain envelope (which we denote by m-m; see Fig. 2B), (3) a song with twice the *D. melanogaster* mean-gain envelope (m-2m; see Fig. 2B), and (4) a silence control condition, in which we did not play back a song. We found that *D. melanogaster* CS females preferred their strain's gain over both flatter gain and steeper gain. The *P*-value is *P*<0.001, where we use a Wilcoxon rank-sum tests (WR); see Fig. 2B.

Other playback studies have used artificial songs for copulation assays (Bennet-Clark and Ewing, 1969; Vaughan et al., 2014), so we examined whether *D. melanogaster* females can differentiate amplitude modulations when they are applied to artificial *D. melanogaster* songs. To test this, we conducted an experiment analogous to the one that we described in the above paragraph, where the only difference is that we applied amplitude envelopes to artificial *D. melanogaster* songs rather than to song recordings of *D. melanogaster* males.

We again found that females preferred their strain's mean gain over both flatter and steeper gains (the *P*-value is *P*<0.001, using a WR; see Fig. 2C), suggesting that females robustly exhibit a bandpass-like preference for their own strain's mean gain. Interestingly, copulation data in Fig. 2B and C seem to have a bimodal distribution, with most couples mating infrequently but a few mating frequently. The origin of this bimodal distribution is not clear, and further work is necessary to study the effects of stimuli and experimental setups on the mating behaviour of *D. melanogaster*.

### Amplitude modulation does not affect other song characteristics

Modulating amplitude in *D. melanogaster* pulse songs may affect other song characteristics, such as IPI, pulse frequencies, cycles per pulse (CPP), or pulse shape (see Fig. 3). If true, the differences in mating that we observed may be due to song characteristics other than amplitude modulation. To test this, we measured and compared song characteristics in our playbacks, and we found that amplitude modulation does not significantly affect these other song characteristics (see Fig. 3). This suggests that the observed differences in behavioural responses were due to modulation of amplitude.

### *D. melanogaster* females respond to small-amplitude pulses

When applying amplitude envelopes to fly songs, some pulses are smaller than others (see Fig. 4A). *D. melanogaster* may not hear small-amplitude pulses (Yoon et al., 2013), and this may affect mating responses. To ensure that flies can hear small-amplitude pulses in playbacks, we controlled for the audibility of pulses in modulated songs (see Fig. 4). To test whether flies can hear pulses with the minimum amplitude in songs (see the ‘Stimulus design’ section in the Materials and Methods), we generated an artificial song in which we set all pulses to the minimum amplitude and played it back to flies. If the pulse amplitudes are too small for flies to hear, they should respond to such pulses in a similar way as to a song that lacks pulses altogether. In contrast, we found that flies responded significantly more to a song with small-amplitude pulses than to a song that lacks pulses (see Fig. 4B,C), which demonstrates that the small-amplitude pulses in our playbacks were processed by the flies. We also found that flies respond more to m-m playbacks that have a larger mean amplitude than to playback with small-amplitude pulses. (The *P*-value is *P*<0.001 in a WR.) This finding agrees with prior observations that auditory responses in flies are sensitive to playback amplitude (Yoon et al., 2013).

We observed that flies responded significantly less to songs that lack pulses even when they included sine song. This agrees with previous findings that showed that flies do not respond immediately to sine song (Yoon et al., 2013; von Schilcher, 1976a). Interestingly, Shirangi and colleagues observed that females mated less frequently with males whose hg1 motor neuron was genetically blocked (which is believed to inhibit sine-song production) (Shirangi et al., 2013). This indicates that lack of sine song affects mating, but it does not demonstrate that sine song necessarily promotes mating; and other studies have suggested that it does not (Yoon et al., 2013; von Schilcher, 1976a).

### Amplitude modulations are prevalent across labs

**Fig. 5.**
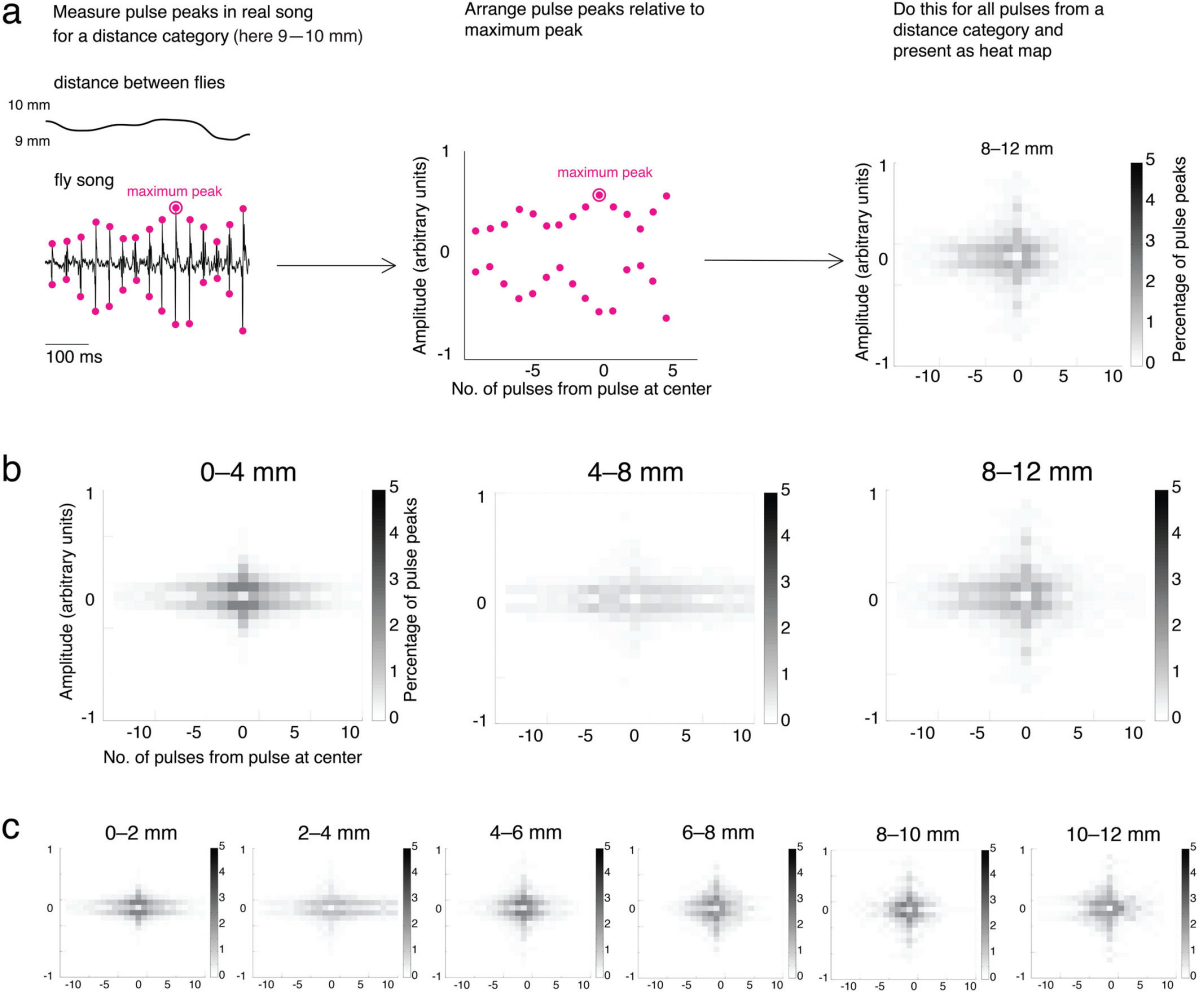
**Distance-independent variations in song amplitude are prevalent in the data of** **Coen et al. (2016****).** Because distance affects song amplitude but not all variation in song amplitude can be explained by distance (Coen et al., 2016), we search the data of Coen et al. (2016) for consecutive pulse trains at specified distances between male and female flies to investigate residual amplitude variation. (A) To analyse residual amplitude variation, we (1) measure pulse peaks for a distance category, (2) arrange consecutive pulse trains of a given distance interval around the pulse peak with maximal amplitude, and (3) plot the arranged peaks as heat maps that illustrate residual variation of pulse amplitude at a given distance interval. We observe residual amplitude variation in the spread of amplitudes along the vertical axis of the heat plots. In panel B, we consider distance intervals of 4 mm. There are *N* pulses at the specified distance ranges. The numbers of pulses are *N*=1376 for 0–4 mm (including both 0 mm and 4 mm), *N*=1420 for 4–8 mm (not including 4 mm), and *N*=985 for 8–12 mm (not including 8 mm). In panel C, we consider distance intervals of 2 mm. The numbers of pulses are *N*=1376 for 0–2 mm (including both 0 mm and 2 mm), *N*=1523 for 2–4 mm (not including 2 mm), *N*=969 for 4­–6 mm (not including 4 mm), *N*=789 for 6–8 mm (not including 6 mm), *N*=575 for 8–10 mm (not including 8 mm), and *N*=500 for 10–12 mm (not including 10 mm).

It is not trivial to accurately measure pulse amplitudes, and Coen et al. went to great efforts to attempt to normalize amplitude measurements (Coen et al., 2016). Because we were interested in whether these normalized measurements exhibit amplitude modulations that are similar to the ones that we observed, we contacted Coen et al., and they kindly supplied us with their measurements. When analysing Coen et al.’s data, we find that distance-independent modulations in song amplitude are prevalent in their data (see Fig. 5). We thus conclude that distance-independent amplitude modulations are prevalent across their and our labs.

## DISCUSSION

Our work demonstrates that flies respond behaviourally to amplitude modulations in their courtship song, suggesting that *Drosophila* can auditorily process amplitude modulations. Mating-call amplitude is processed throughout the animal kingdom: the mating decisions of mammals (Clutton-Brock and Albon, 1979), birds (Ritschard et al., 2010), amphibians (Velez, 2013), and other insects (Roemer, 2013) are all influenced by song amplitude. Our study demonstrates that the mating decisions of the widely used model organism *D. melanogaster* (Jennings, 2011) are also influenced by song amplitude. It will be interesting to explore neural mechanisms for auditory processing of amplitude modulations in *D. melanogaster*, just as it is interesting to investigate neural mechanisms for processing song characteristics such as IPI (Vaughan et al., 2014; Zhou et al., 2014, 2015) and frequency (Kamikouchi et al., 2009).

Perceived amplitude can be affected by noise (Samarra et al., 2009), and positional changes during courtship can also affect song perception (Morley et al., 2012). Positional changes are frequent during courtship: when singing, a male circles a female, sometimes singing in front of her and sometimes singing behind her. Morley et al. showed that females cannot hear a song when the song source comes from behind their back (Morley et al., 2012). This implies that some parts of a song may be occluded from female perception due to movement. Perceived amplitude is likely distorted because of several factors, including the position of a singer (Morley et al., 2012), rapid attenuation of perceived amplitude (Tauber and Eberl, 2003), male–male competition (Tauber and Eberl, 2002), and environmental noise sources such as wind (Samarra et al., 2009). Therefore, it is informative to examine whether flies can cope with noisy song amplitude structures.

Our work shows that *D. melanogaster* behaviourally discriminate songs with differently-shaped amplitude masks. (See the Materials and Methods section for more details on amplitude-mask generation.) The masks that we used in our study are diamond-like (see Fig. 1B), and we demonstrated that flies can discriminate these songs from songs with constant-amplitude rectangular-like masks, suggesting that flies may be able to process differences in the shapes of amplitude masks. It will be interesting to test this possibility in future experiments.

We have observed amplitude modulations in pulse songs, and others have reported them as well (Coen et al., 2016; Ewing, 1977). It will also be interesting to further investigate amplitude modulations in sine song (Arthur et al., 2013). Studying sine song is difficult, because it has a low mean intensity (Ewing, 1977; von Schilcher, 1976b); and automated and manual methods for sine-song detection yield different results (Coen et al., 2016; Arthur et al., 2013). Therefore, a comprehensive analysis of sine-song amplitude modulation will require improvements both in recording techniques and in song-analysis software.

It is not obvious what methods to use to analyse patterns in noisy biological data such as fly-song amplitude, and studies of fly songs will benefit from further methodological developments (Arthur et al., 2013; Kyriacou and Hall, 1980; Stern, 2014; Kyriacou et al., 2017). We measured amplitude gain, and it may be possible to also investigate song amplitude structure using spectral analysis (Stern, 2014; Kyriacou et al., 2017; Stern et al., 2017).

Our work demonstrates that changes in either song amplitude structure or IPI affect mating behaviour (see Fig. 2). IPI is well-established in the study of *Drosophila* songs, and it has been suggested that IPI conveys species-specific information (Bennet-Clark and Ewing, 1969; Ritchie et al., 1999; Yoon et al., 2013). IPIs differ between species, and females prefer songs with their species-specific IPI (Bennet-Clark and Ewing, 1969; Ritchie et al., 1999; Yoon et al., 2013). It has been demonstrated that constant-length IPIs have little effect on female behaviour in the important *melanogaster*–*simulans* range (35–45 ms) (von Schilcher, 1976a). We find that the IPIs that we study (3 ms and 73 ms), which are smaller and larger than those in the *melanogaster*–*simulans* range, robustly yield different behaviours in flies.

Interestingly, when IPIs change gradually, flies appear to be able to distinguish between smaller differences in IPIs in the *melanogaster*–*simulans* range (Ritchie et al., 1999). From studying IPIs, it is known that flies possess auditory circuits that act as bandpass filters for their species IPIs (Vaughan et al., 2014; Zhou et al., 2014, 2015). It will be interesting to examine whether flies can analogously process song amplitude structure. For example, it has been reported that different *D. melanogaster* strains vary in their IPIs (Arthur et al., 2013), and future investigations can examine whether song amplitude structure differs across strains or across species. Additionally, circadian-rhythm mutations (such as *period*) affect IPI (Kyriacou and Hall, 1980) and substrate-borne vibrations (Medina et al., 2015), and such mutations may also affect song amplitude structure. Notably, amplitude modulation can extend over long timescales (Coen et al., 2016), which may help explain why female flies seem to process auditory information about male courtship song over such timescales (Clemens et al., 2015). It is worth investigating potential links between long-timescale auditory processing and amplitude modulations.

## MATERIALS AND METHODS

### Flies and experimental protocols

We grew *D. melanogaster* Canton-S flies at room temperature in a 12 h light–dark cycle on a standard cornmeal-based medium in a temperature-controlled room, with a set temperature of 25°C. We collected male and female virgin flies within six hours after eclosion. In playback experiments, we controlled sound levels throughout playback with a CEL-246 sound-level meter (Casella, Buffalo, NY, USA). Because we used playback stimuli with variations in amplitude, sound levels varied between about 85 dB and about 100 dB. We measured sound levels with the sound-level meter positioned at the centre of our speaker, with a distance of 10 cm to the speaker. We controlled for mean playback amplitude by generating playback stimuli that have similar mean amplitudes. (There was less than a ±1% amplitude difference between playbacks.) We recorded songs of 15 males, who we paired with females from the same strain. We recorded songs for 10 min.

Before song recording and playback experiments, we aged males in isolation for 3–7 days, and we aged females in groups of eight animals of the same genotype for 3–7 days. For song recordings, we placed one male and one female in a cylindrical mating chamber of 10 mm diameter and 4 mm height. The floor of each mating chamber was a plastic grid that allows air vibrations to pass to the microphone. We paired couples of the same species and strain, and we recorded songs throughout the day.

For copulation assays, we muted and deafened male flies by cutting their wings and arista, respectively (Bennet-Clark and Ewing, 1969). Male flies in the copulation assay were thus unable to hear song playback, whereas females were able to hear it. The copulation assay is thus relevant for female responses to songs; deafened males do not respond to songs (Inagaki et al., 2010). We conducted copulation experiments throughout the day, and we did not use blinding for analysing playback assays.

We recorded songs with a CMP-5247TF-K particle-velocity microphone (Arthur et al., 2013) in a fly-song box (http://sine.ni.com/cs/app/doc/p/id/cs-17145?nisrc=RSS-featured-en\#). In line with prior experiments (Peixoto and Hall, 1998; Chakravorty et al., 2014; Bernstein et al., 1992; Blyth et al., 2008; Menezes et al., 2013), we recorded songs with a single microphone and a male singing on top of this microphone. We controlled for distance to the microphone by using a setup in which a male was always on top of the microphone, instead of one in which a male can distance himself from it (Coen et al., 2016, 2014; Clemens et al., 2015). Microphone sensitivity can vary across a microphone (http://www.datasheetlib.com/datasheet/902008/cmp-5247tf-k_cui.html), so the position of a male on a microphone can affect the recorded song amplitude. A limitation of our study is that we do not have data on the positions of males and thus cannot control for such positional effects.

We played back songs with a Mach sub-bass speaker, which was connected to an EP-800 amplifier (Prolight Concepts Group, Darwen, UK), which in turn was connected to a Creative sound blaster X-Fi Xtreme audio PCI sound card (Creative, Singapore, Singapore) in an OptiPlex 3020 mini tower PC (Dell, Round Rock, USA). We used Windows Media Player (version 12.0.7601.19148) with the default setting for all playbacks. We placed flies for playback experiments in chambers with rectangular cross sections. They are similar to the chambers described in Inagaki et al. (2010), although ours have a plastic grid, instead of nylon mesh, for the front and back walls. After placing flies in our playback chambers, we positioned them on a box of 30 cm height to level the flies at the speaker centre. We illuminated the flies from below with LED lights in a size-A4 comic master light table.

### Data analysis

We automatically segmented recorded songs with FlySongSegmenter (Arthur et al., 2013). This song-analysis methodology was criticized recently (Kyriacou et al., 2017), but we found that FlySongSegmenter detected about 95% of the pulses that we detected manually. Additionally, FlySongSegmenter is used in the fly-song community as software for song analysis (Coen et al., 2016, 2014; Stern, 2014; Clemens et al., 2015). In a recent study, it was observed that FlySongSegmenter successfully detected only about 50% of pulses that were detected manually (Kyriacou et al., 2017). However, when those data were reanalysed by Stern et al. (2017 preprint), he found that FlySongSegmenter detected about 80% of the manual-detected pulses. Both Kyriacou et al. (2017) and Stern et al. (2017) compared the same songs and used the same manual annotations, suggesting that the differences in accuracy are not due to those factors. Additionally, FlySongSegmenter allows users to adjust settings, and we observed that these settings can strongly affect pulse detection. We thus make our settings available on Figshare (https://doi.org/10.6084/m9.figshare.5923573). The accuracies that were reported originally for detecting pulses with FlySongSegmenter lay between 80% and 99% (Arthur et al., 2013), and our accuracies also lie in this range. After song segmentation, we examined amplitude gain. Gain is the increase in amplitude per second, and amplitude indicates the maximum peak of a pulse. Our analysis scripts (in Matlab) are available on Figshare (https://figshare.com/s/d42b0cfe865011e5be0906ec4bbcf141). We now outline our analysis workflow:

1. Calculate the amplitude maxima max(*P_i_*) of individual pulses, where *P_i__ _*is the *i*th pulse identified by FlySongSegmenter in a song recording. The time *t_i_*_ _is the time in milliseconds from the start of the song recording to the time that achieves max(*P_i_*).
2. Calculate the slope as the proportional amplitude change per time. The amplitude slope is

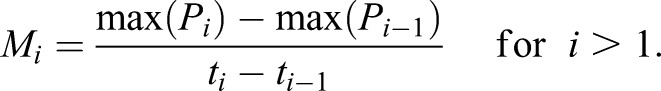

In our study, we focused on gain and thus only examined increasing slopes (i.e. slopes with *M_i_ *> 0).

We manually counted the number of copulating couples during 20 min of playback. Our fly-copulation data and our code for analysing these data are available on Figshare (https://figshare.com/s/a6535e5fc86ba4d74d9b). Our two-sample comparisons were two-sided Wilcoxon rank-sum tests (WR), as the sample distributions are non-normal. When appropriate, we corrected for multiple testing using Bonferroni correction. Additionally, we show log-rank tests for comparing survivorship curves in Fig. 2. The results of the log-rank tests agree with the results of our other statistical computations, including the Wilcoxon rank-sum tests and ANOVAs, so we are confident that the differences that we report in our data are statistically robust. In our experiments, we compared conditions that include song playback with silence. Silence is an important control condition, though it may not provide evidence for heterogeneity of treatments that include songs. Therefore, we computed ANOVAs to compare only song conditions. For each condition, we conducted multiple trials; in each trial, we observed 12 couples and examined the number of couples that were copulating every minute for 20 min. We give the number of trials for each experiment and each condition: experiment (1) concerns the impact of IPI on copulation rates, experiment (2) concerns the impact of song amplitude structure in song recordings on copulation rates, and experiment (3) concerns the impact of song amplitude structure in artificial songs on copulation rates. Experiment (1) included three song conditions: (1.1) IPI = 3 ms, (1.2) IPI = 38 ms, and (1.3) IPI = 73 ms. The numbers of trials were *n_t_*=5 for (1.1), *n_t_*=5 for (1.2), and *n_t_*=5 for (1.3). Experiment (2) included three song conditions: (2.1) flat gain, (2.2) *D.* *melanogaster* song with *D.* *melanogaster* gain (m-m), and (2.3) *D.* *melanogaster* song with twice the *D.* *melanogaster* gain (m-2m). The numbers of trials were *n_t_*=9 for (2.1), *n_t_*=10 for (2.2), and *n_t_*=9 for (2.3). Experiment (3) included the same three song conditions as those for experiment (2). The numbers of trials were *n_t_*=4 for (3.1), *n_t_*=10 for (3.2), and *n_t_*=4 for (3.3). We computed ANOVAs for survival curves of copulation rates over 20 min. Thus, each trial consists of 20 data points, corresponding to the 20 min that we observed couples. The number of degrees of freedom (dof) is a function of the number of trials (*n_t_*), the number of minutes per trial (*n*_m_), and the number of song conditions (*n*_sc_). It is 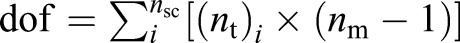. For experiment (1), we find that *F*≈34.51 (where *F* is the value computed by an ANOVA *F* statistic, which shows if a group of variables are jointly significant) with a *P*-value of *P*<0.0001 and dof = 299; for experiment (2), *F*≈10.99, *P*<0.0001, and dof = 559; and for experiment (3), *F*≈18.33, *P*<0.0001, and dof = 359. To assess the relative effect size of each experiment, we compute *Cohen's* *f*, which we denote by *C_f_*, for the ANOVAs: 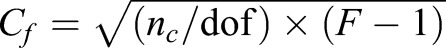. For experiment (1), we find that *C_f_* ≈ 0.47; for experiment (2), *C_f_* ≈ 0.19; and for experiment (3), *C_f_* ≈ 0.31. The effect size is thus large for experiment (1) and medium for experiments (2) and (3) (http://imaging.mrc-cbu.cam.ac.uk/statswiki/FAQ/effectSize).

### Stimulus design

For the experiments that we presented in Figs 2 and 4, we designed playback stimuli from recordings of vigorous courtship song of *D. melanogaster*. See Fig. 1 for stimuli design, and see Fig. 3 for playback statistics. For the experiment that we presented in Fig. 2B, we modulated amplitude in an artificial *D. melanogaster* song generated by Joerg Albert (Ear Institute, University College London).

We controlled the mean amplitude of playback stimuli by computing the mean amplitude of each playback stimulus and subsequently adjusting the mean amplitudes to differ from each other by less than ±1%. We did this by identifying the playback *p_A_* with the largest mean amplitude *A* of playbacks in an experimental condition and then calculating the relative difference Δ*A = A*/*a_i_* in mean amplitude, where *a_i_*_ _is the mean amplitude of the *i*th playback *p_i_*_ _= *p_i_*(*t*) in an experimental condition. Additionally, *p_i_*(*t*) is a time series, where *t* is time, which we discretize in units of 1/3000 of a second. We then multiplied playback *p_i_*(*t*) by Δ*A*, so *p*_Δ_(*t*) = *p_i_*(*t*)×Δ*A*.

Our Matlab code for generating playback stimuli from *Drosophila* audio tracks is available on Figshare (https://figshare.com/s/fb5f8110865011e5b0ef06ec4b8d1f61). We now outline our workflow:

1. Detect pulses 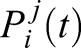 with FlySongSegmenter (Arthur et al., 2013), where the subscript *i* identifies the pulse and the superscript *j* denotes which song is being analysed.
2. Normalize pulse amplitudes to generate a piecewise-constant amplitude audio track 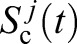. Pulse amplitude maxima and minima can have different absolute values, and we set them to the same absolute value after normalization. We normalized maxima and minima separately in two steps. For normalizing maxima, we (1) determined the pulse maxima 
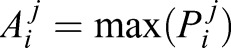
. We then (2) split the audio track *S*^*j*^(*t*) along the horizontal axis to generate split audio tracks – one with the positive-amplitude portions and the other with the negative-amplitude portions – to separately normalize the amplitude maxima and minima. We generated a split audio track 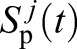 that consists of all nonnegative *S*^*j*^(*t*). (3) We normalized the pulses in the split audio track 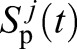 by dividing them by their pulse-amplitude maximum 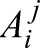. For 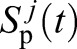, we set all of the negative parts equal to 0. To (4) normalize the pulse-amplitude minima, we determined the pulse minima 
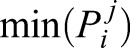
. We then (5) split the audio track *S*^*j*^(*t*) along the horizontal axis to generate 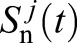, which consists of all negative *S*^*j*^(*t*), and (6) normalized pulses in the split audio track 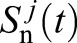 by dividing them by their amplitude minimum 
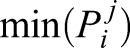
. For 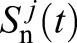, we set all of the positive parts equal to 0. Finally, we (7) joined the normalized split audio tracks 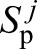 and 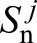 to generate a piecewise-constant-amplitude audio track 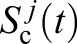, such that 
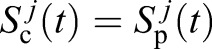
 for all *t* that satisfy *S*^*j*^(*t*) ≥ 0 and 
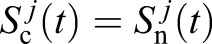
 for all *t* that satisfy *S*^*j*^(*t*) < 0.
3. Make amplitude masks as functions of amplitude gain. We introduce the term ‘amplitude mask’ to describe amplitude-modulated soundtracks of fly pulse song. Because masks with 0 values can set pulse amplitudes to 0 and hence effectively erase pulses, we allow masks to take values between 0.2 and 1 (i.e. the mask *m*_m_ ∈ [0.2, 1]), as we find that flies respond to pulses when we set their amplitudes to 0.2 (see Fig. 4).
  We generate masks *m*_m_ such that

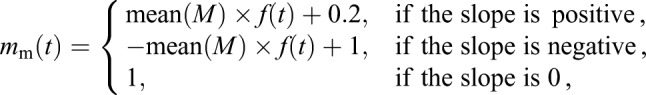
where *M* is the measured gain in fly-song recordings of a particular strain. The function *f*(*t*) gives a discrete linear increase of duration *d*. Specifically, 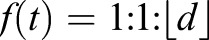 (using Matlab notation; thus, the sequence is 1, 2, 3, 4,…, *d*), where *d* = 0.8/mean(*M*) is the duration of increase from 0.2 to 1, the term mean(*M*) denotes the mean gain, and 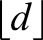 is the integer part of *d*. The masks that we used in our study are symmetric along their maxima, so the increasing and decreasing slopes have equal absolute values. See Fig. 1B for an illustration of the amplitude masks. We started a new envelope at the beginning of each pulse train. As also done by other authors (Arthur et al., 2013; Coen et al., 2014), we defined pulse trains as consecutive trains of pulses with a duration of 300 ms or more to adjacent pulse trains.
  The choice of a temporal gap of 300 ms is arbitrary, and one can choose a different duration. When one makes a choice for what duration to expect between trains of pulses, one should consider whether this choice results in more, fewer, or (roughly) the same number of pulse trains as one would detect manually. The choice of a gap of 300 ms duration results in no significant difference in the numbers of pulse trains that one detects either automatically or manually (Arthur et al., 2013).

4. Mask the constant-pulse audio track *S*_c_(*t*) with gain functions *m*_m_(*t*) and thereby generate a masked audio track *S*_m_(*t*) = *S*_c_(*t*)×*m*_m_(*t*).

The only difference between the playback signals were the amplitude envelopes; the other features of recorded songs were the same (see Fig. 3). Following other authors (Peixoto, and Hall, 1998; Menezes et al., 2013; Partridge et al., 1987), we measured pulse amplitude as the maximum of the pulses. An alternative measure of amplitude is peak-to-peak amplitude, which is the difference between the maximum and minimum peak of a pulse and indicates the maximum signal that a female can detect (Coen et al., 2016). To test whether the choice of measuring maximum amplitude versus peak-to-peak amplitude makes a significant difference, we examined the Pearson correlation of the two measures in our song recordings and found that they were significantly positively correlated (*R*^2^ ≈ 0.96, with a *P*-value of *P*<0.0001), which suggests that maximum amplitude and peak-to-peak amplitude are comparable.

## Supplementary Material

First Person interview
