# Supplementary material for: Female Drosophila melanogaster respond to song-amplitude modulations
Source: Biol Open. 2018 Apr 17;7(6):bio032003. doi: 10.1242/bio.032003 (PMC6031343; doi:10.1242/bio.032003)
Supplement: First Person interview [file biolopen-7-032003-s1.pdf]

© 2018. Published by The Company of Biologists Ltd | Biology Open (2018): doi:10.1242/bio.032003: Supplementary information

FIRST PERSON

First person – Birgit Brüggemeier

First Person is a series of interviews with the first authors of a selection of papers published in Biology Open, helping early-career researchers promote themselves alongside their papers. Birgit Brüggemeier is first author on 'Female *Drosophila melanogaster* respond to song-amplitude modulations', published in BiO. Birgit is a research associate in the lab of Bernd Edler at Fraunhofer-Institut für Integrierte Schaltungen, Erlangen, Germany, investigating audio communication signals, how they are produced, and how they are perceived and processed.

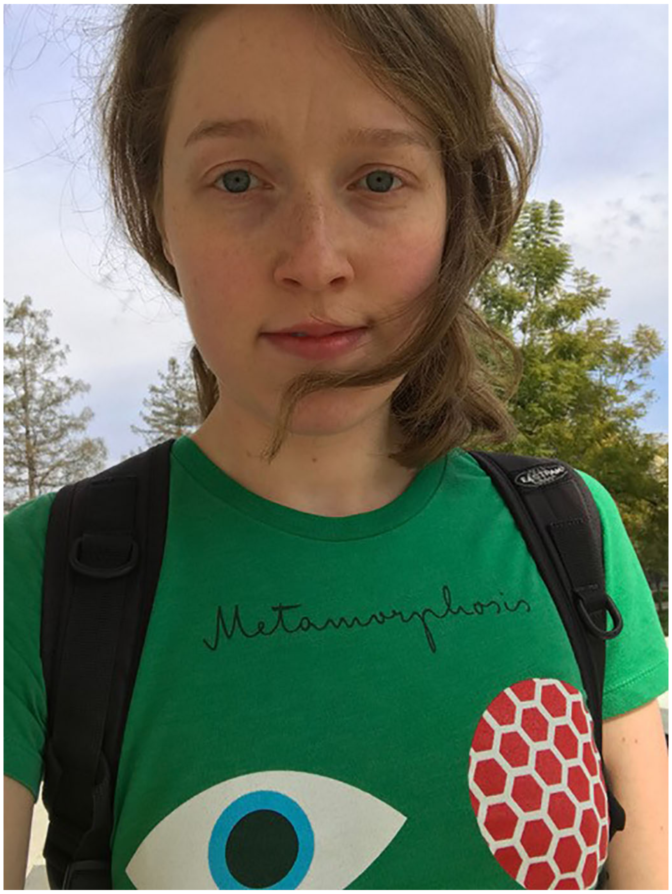

Birgit Brüggemeier

What is your scientific background and the general focus of your lab?

My studies have been as varied as the topic they revolve around: communication. I started off studying English literature and linguistics as a part-time university student during high school. For my bachelor's degree, I began studying media and communications, but decided to switch to psychology after taking a couple of very interesting classes in that subject. Then I applied for and was accepted onto a 4-year PhD programme in neuroscience funded by the Wellcome Trust. My PhD studies took place in Stephen Goodwin's lab at the University of Oxford, focusing on the courtship song of the fruit fly *Drosophila melanogaster*, and ventured to model fly song production as well, co-supervised by Mason A. Porter, a professor of applied mathematics at UCLA. After completing my PhD, I started a postdoc at AudioLabs at the Fraunhofer Institute where I now communicate with machines instead of flies, as we develop speech assistance devices.

How would you explain the main findings of your paper to non-scientific family and friends?

You may know fruit flies as unwanted visitors in your kitchen. They are small and we tend to ignore them or want to get rid of them; however, for scientists they are a favourite research subject. One reason for this is their complex communication behaviour. Fruit flies court, and as part of their courtship they produce songs by vibrating their wings. These songs are structured, and flies process the songs they hear to make mating decisions, i.e., "Do I like the singer or not?" In our research we describe a novel song characteristic in fly song, which we call song amplitude structure (SAS). We show that SAS influences mating decisions of the fruit fly *D. melanogaster*.

What are the potential implications of these results for your field of research?

Song amplitude structure is a novel characteristic in fly song, and we first describe it in the fruit fly *D. melanogaster*, which is one of hundreds of fruit fly species. Further work may look into other species and investigate if flies use SAS as a species-specific signal to discriminate between viable partners. In addition, it will be interesting to investigate how flies produce SAS and how they perceive it on a physiological level. There have been numerous studies looking into physiological mechanisms of song production and perception, and SAS is a new song characteristic to study in this way.

"In a nutshell, my research made me humbly in awe of flies."

What has surprised you the most while conducting your research?

In a nutshell, my research made me humbly in awe of flies. A lot of my research involved behavioural experiments, which I used to determine fly reactions to subtle differences in their song – differences that were so subtle I wasn't able to perceive them, but flies often were.

In your view, what are important issues in science politics?

My views on science politics are shaped by my background, which is coming from a migrant family and being a female working in STEM. I was born in Romania before the iron curtain fell and the USSR fell apart. My family fled Romania when I was two years old. Both of my parents were trained academics, my father an engineer and my mother a teacher. Coming to Germany their degrees were not recognised and they changed careers. This was almost 30 years

Birgit Brüggemeier's contact details: AudioLabs, Fraunhofer-Institut für Integrierte Schaltungen, 91058 Erlangen, Germany.

E-mail: birgit@brueggemeier.net

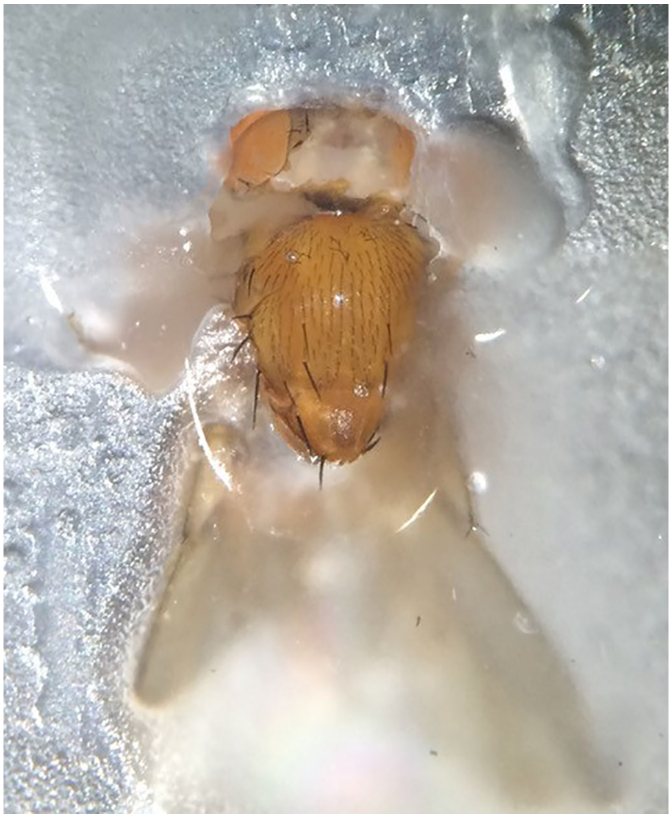

Neuroscience in the fly – living fly on plastic slide with exposed brain.

ago, before globalisation became a buzz-word. Today I teach students from all over the world and from them I hear stories that are not unlike my parents' stories; degrees are not recognised, careers are changed, potential is lost. As a science community it is in our interests to preserve potential and stand up for systems that make achievements comparable across the world.

As a woman doing research in STEM, I have been mistaken for a secretary on numerous occasions when starting research jobs. I believe this does not result from misogyny, but reflects gender distributions across job roles; researchers in STEM are predominantly male and secretaries are predominantly female. I believe there are many high-potential young women who are derailed by merely observing this fact. A former professor of mine, Eva Bamberg, studied gender gaps in STEM and her research suggested that next to institutional biases, individual biases can cement gender inequality. Female researchers report more self-doubt than their male colleagues and promote themselves less than their male counterparts. Institutional biases are slow to change and becoming aware of biases within ourselves is a first step towards changing them.

> **"Institutional biases are slow to change and becoming aware of biases within ourselves is a first step towards changing them."**

Who are your science heroes?

Alan Turing was a brilliant mathematician who is well-known for helping to end WW2 by cracking Nazi codes. He is less renowned for his reaction–diffusion model, which helps to explain complex biological patterns, like spots on cows or growth patterns of moulds. Turing's work on reaction–diffusion changed my conception of patterns in fly song and inspired some of my work, which is why he won a place in my heart and became one of my science heroes. Turing's work follows me in my new role designing speech assistance devices in the form of the 'Turing test', which he proposed as means of testing if machines are human-like. More than 60 years after his death, we have not been able to develop machines that pass the Turing test, so Turing continues to challenge me.

Nikolaas Tinbergen was an intent observer and was honoured for his work in ethology with the Nobel prize. Biology textbooks showcase his work on the courtship dance of the stickleback and tell the story of how he tricked seagulls into neglecting their eggs. I see in his research a willingness to neglect human perspective and to focus on the communication system he investigates. I believe his lack of anthropomorphism allowed him to uncover beautiful oddities, like the seagull mother who would neglect her eggs in favour of a giant speckled ball. In my research with flies I learned that my human perspective does not help and sometimes hinders my understanding of their communication, and so Tinbergen became my role model in his relentless focus on the animals he studied. As I now study human–computer interactions and attempt to make machines more human-like, my current focus happens to be on humans and it comes in handy to be human at last.

Reference

Brüggemeier, B., Porter, M. A., Vigoreaux, J. O. and Goodwin, S. F. (2018). Female *Drosophila melanogaster* respond to song-amplitude modulations. *Biol. Open* **7**: bio032003.
